# Long-Range Azimuthal Correlation, Entanglement, and Bell Inequality Violation by Spinning Gluons at the Large Hadron Collider

**DOI:** 10.34133/research.0552

**Published:** 2025-02-05

**Authors:** Yuxun Guo, Xiaohui Liu, Feng Yuan, Hua Xing Zhu

**Affiliations:** ^1^ Nuclear Science Division, Lawrence Berkeley National Laboratory, Berkeley, CA 94720, USA.; ^2^ Center of Advanced Quantum Studies, School of Physics and Astronomy, Beijing Normal University, Beijing 100875, China.; ^3^ Key Laboratory of Multi-scale Spin Physics, Ministry of Education, Beijing Normal University, Beijing 100875, China.; ^4^ Institute for Theoretical Physics, Universität Tübingen, D-72076 Tübingen, Germany.; ^5^ School of Physics, Peking University, Beijing 100871, China.; ^6^ Center for High Energy Physics, Peking University, Beijing 100871, China.

## Abstract

We apply the recently developed concept of the nucleon energy–energy correlator (NEEC) for the gluon sector to investigate the long-range azimuthal angular correlations in proton–proton collisions at the Large Hadron Collider. The spinning gluon in these collisions will introduce substantial nonzero cos(2ϕ) asymmetries in both Higgs boson and top quark pair productions, where ϕ is the azimuthal angle between the forward and backward energy correlators in the NEEC observables. The genesis of the cos(2ϕ) correlation lies in the intricate quantum entanglement. Owing to the substantial cos(2ϕ) effect, the NEEC observable in Higgs boson and $t\bar{t}$ production emerges as a pivotal avenue for delving into quantum entanglement and scrutinizing the Bell inequality at high-energy colliders.

## Introduction

Long-range correlation in particle productions in proton–proton (pp) collisions at the Large Hadron Collider (LHC) has attracted great attention in the last decade with tremendous efforts from both experiment and theory sides [1–4]. In this paper, we investigate this physics from a different perspective, applying the nucleon energy–energy correlator (NEEC) [5–7] at the LHC. We will show that the spinning gluon distribution in this framework [7] leads to sizable $\text{cos}\left(2\phi \right)$ azimuthal asymmetries in forward–backward energy correlators in pp collisions, where ϕ is the azimuthal angle difference between these two energy correlators. These long-range $\text{cos}\left(2\phi \right)$ asymmetries are signatures of the quantum entanglement, thereby providing the first test of the Bell inequality [8,9] within the entangled gluon system. Pursuing such a test in the Standard Model (SM) of particle physics at high-energy colliders has been very active in recent years [10–33]. In particular, exciting observations of quantum entanglement in top quark pair production in pp collisions at the LHC have been reported by the ATLAS and CMS collaborations [34–36].

The NEEC was introduced by Liu and Zhu [5] as a new method to explore the nucleon structures. It employs an asymptotic energy flow operator $\hat{E}(\theta_{a})$, which measures energy deposits in the detector at a fixed angle $\theta_{a}$ relative to the nucleon incoming beam direction in collider experiments. Previous studies mainly focused on deep inelastic scattering [5–7,37], which will be explored at the future electron–ion collider [38–40]. In the following, we will study the NEEC observables in pp collisions. The comparison between these two collision systems will provide an opportunity to test the universality of NEECs. Meanwhile, the novel phenomena unveiled below will stimulate further experimental investigations and help decipher the origin of nearside ridges in pp collisions.

**Fig. 1.**
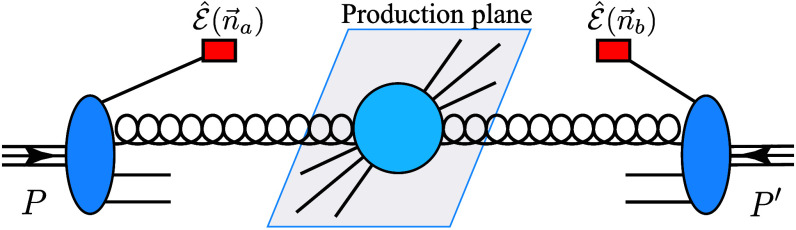
Nucleon energy–energy correlator measurements in proton–proton collisions at the Large Hadron Collider (LHC). Energy deposits in the forward directions of both incoming hadron beams with polar angles $\theta_{a,b}$ and azimuthal angles $\phi_{a,b}$ represented by ${\vec{n}}_{a,b}$, respectively.

To investigate the NEEC at the LHC, we propose to measure the energy deposits along the beam directions of incoming hadrons with polar angles $\theta_{a,b}$ and azimuthal angles $\phi_{a,b}$, respectively; see the illustration in Fig. 1. The hard partonic scattering produces, e.g., the Higgs boson or top quark pairs. The experiment can be carried out by a coincidence measurement between the forward/backward energy flows and the hard interactions in the center. Because $\theta_{a}$ and $\theta_{b}$ are either small or close to π and in opposite directions, their rapidity difference will be large, for which we refer to as a long-range correlation. Meanwhile, we will show that different processes lead to different $\text{cos}\left(2\phi \right)$ asymmetries. In particular, we find that the asymmetries in Higgs boson and top quark pair productions are quite sizable but with opposite signs. Therefore, a detailed study of these correlations will open a new avenue for precision SM physics.

In the following, we focus on the gluon NEEC [7]:

$$\begin{array}{c} \begin{array}{c} f_{g,\mathrm{EEC}}^{\alpha \beta }\left(x,\;{\vec{n}}_{a}\right)=\int \frac{{\mathrm{dy}}^{-}}{2\pi {\mathrm{xP}}^{+}}e^{-{\mathrm{ixP}}^{+}y^{-}}\\ \times \langle P|F^{+\alpha }\left(y^{-}\right)L^{†}\left[\infty ,\;y^{-}\right]\hat{E}\left({\vec{n}}_{a}\right)L\left[\infty ,\;0\right]F^{+\beta }\left(0\right)|P\rangle \\ =\left(-g_{T}^{\alpha \beta }/2\right)f_{g,\mathrm{EEC}}\left(x,\;\theta_{a}^{2}\right)+h_{T}^{\alpha \beta }d_{g,\mathrm{EEC}}\left(x,\;\theta_{a}^{2}\right), \end{array} \end{array}$$ (1)

for the proton moving in the $+\hat{z}$ direction with momentum *P*, where F is the gauge field strength tensor and L is the gauge link. We have kept the azimuthal dependence of the energy flow direction $\begin{array}{c} n_{a}^{\alpha }=\left(1,\;\text{sin}\theta_{a}\;\text{cos}\phi_{a},\;\text{sin}\theta_{a}\;\text{sin}\phi_{a},\;\text{cos}\theta_{a}\right) \end{array}$. To parameterize the spinning gluon distribution, we introduce two projection tensors: $g_{T}^{\alpha \beta }=g^{\alpha \beta }-\left(P^{\alpha }{\bar{n}}^{\beta }+{\bar{n}}^{\alpha }P^{\beta }\right)/\bar{n}\cdot P$ and $h_{T}^{\alpha \beta }=n_{a,T}^{\alpha }n_{a,T}^{\beta }/\left|n_{a,T}^{2}\right|+g_{T}^{\alpha \beta }/2$, with $\bar{n}\cdot P=P^{0}+P^{z}\equiv P^{+}$ and $n_{a,T}^{\alpha }=\left(0,{\vec{n}}_{a},\;0\right)$ is the transverse component of $n_{a}^{\alpha }$. These two tensors help to define the normal gluon NEEC $f_{g,\mathrm{EEC}}\left(x,\;\theta_{a}^{2}\right)$ and the spinning gluon NEEC $d_{g,\mathrm{EEC}}\left(x,\;\theta_{a}^{2}\right)$, respectively. Similarly, we can define the gluon NEECs for the proton moving in the $-\hat{z}$ direction with momentum $P^{\prime }$ and energy flow direction $\begin{array}{c} n_{b}^{\alpha }=\left(1,\;\text{sin}\theta_{b}\;\text{cos}\phi_{b},\;\text{sin}\theta_{b}\;\text{sin}\phi_{b},\;\text{cos}\theta_{b}\right) \end{array}$. The spinning gluon NEEC $d_{g,\mathrm{EEC}}\left(x,\;\theta_{a}^{2}\right)$ originates from the interference between different helicity states. To generate a long-range correlation between ${\vec{n}}_{a}$ and ${\vec{n}}_{b}$, we need to couple two $d_{g,\mathrm{EEC}}\left(x,\;\theta^{2}\right)$ from both incoming protons, resulting in a $\text{cos}\left(2\phi \right)$ asymmetry, where $\phi =\phi_{a}-\phi_{b}$.

The spinning gluon distributions of the nucleon have also been studied in the literature under different contexts. In the generalized parton distribution (GPD) framework [41–44], the spinning gluon GPD, also called helicity-flip gluon GPD, predicts a $\text{cos}\left(2\phi \right)$ asymmetry in the exclusive processes [45–47]. Meanwhile, in the transverse-momentum-dependent (TMD) formalism, the spinning gluon distribution, referred to as the linearly polarized gluon distribution, leads to a $\text{cos}\left(2\phi \right)$ asymmetry in the associated TMD processes [48–55]. More recently, the $\text{cos}\left(2\phi \right)$ asymmetry has also been discussed in the context of jet substructures [56–60]. The comparison of these measurements will help us understand the quantum chromodynamics (QCD) associated with the spinning gluon.

## Results and Discussion

### NEEC for Higgs boson and top quark pair processes at the LHC

The factorization for NEEC in pp collisions is similar to that for deep inelastic scattering processes [7]. As shown in Fig. 1, we measure the energy flows in 2 arbitrary pixels on the calorimeter located at $\begin{array}{c} {\vec{n}}_{a}=\left(\text{sin}\theta_{a}\;\text{cos}\phi_{a},\;\text{sin}\theta_{a}\;\text{sin}\phi_{a},\;\text{cos}\theta_{a}\right) \end{array}$ and $\begin{array}{c} {\vec{n}}_{b}=\left(\text{sin}\theta_{b}\;\text{cos}\phi_{b},\;\text{sin}\theta_{b}\;\text{sin}\phi_{b},\;\text{cos}\theta_{b}\right) \end{array}$. The polar angles are measured with respect to the *z* axis, i.e., the particular rapidities, and the azimuthal angles are measured from the transverse plane perpendicular to the beam direction. We require each of the two pixels to be much closer to one of the hadron beams. Therefore, these two particles are in opposite directions, forward/backward in the lab frame, e.g., $\theta_{a}\to 0$ and $\theta_{b}\to \pi$. The generic cross-sectional measurement takes the following form:

$$\begin{array}{c} \begin{array}{c} \Sigma \left(Q^{2};\theta_{a,b},\;\phi \right)=\sum_{\mathrm{ij}}\int \mathrm{d\sigma}\left(Q^{2}\right)\frac{E_{i}}{E_{P}}\frac{E_{j}}{E_{P}}F\left(\phi ;{\vec{n}}_{a,b}\right)\\ \times \delta \left({\vec{n}}_{a}-{\vec{n}}_{i}\right)\delta \left({\vec{n}}_{b}-{\vec{n}}_{j}\right), \end{array} \end{array}$$ (2)

where $E_{P}$ represents the beam energy in pp collisions at the LHC and $E_{i}$ and $E_{j}$ represent the energy deposits of particles in ${\vec{n}}_{i}$ and ${\vec{n}}_{j}$ directions, respectively. $F\left(\phi ;\;{\vec{n}}_{a,b}\right)$ imposes the phase space measurement to construct ϕ. In particular, it measures the polar angles $\theta_{a}$ and $\theta_{b}$ along the beam direction for ${\vec{n}}_{a}$ and ${\vec{n}}_{b}$, respectively, and the azimuthal angle difference $\phi =\phi_{a}-\phi_{b}$, where $\bar{\phi }=\left(\phi_{a}+\phi_{b}\right)/2$ is integrated out. In the above equation, $\mathrm{d\sigma}\left(Q\right)$ represents a partonic scattering cross-section. Following previous examples [5], the factorization formula can be written as

$$\begin{array}{c} \begin{array}{c} \Sigma \left(Q^{2};\theta_{a,b},\;\phi \right)\\ =\int d\Omega \left\{x_{a}f_{g,\mathrm{EEC}}\left(x_{a},\;\theta_{a}^{2}\right)x_{b}f_{g,\mathrm{EEC}}\left(x_{b},\;\theta_{b}^{2}\right){\hat{\sigma }}_{0}\right.\\ \left.+x_{a}d_{g,\mathrm{EEC}}\left(x_{a},\;\theta_{a}^{2}\right)x_{b}d_{g,\mathrm{EEC}}\left(x_{b},\;\theta_{b}^{2}\right){\hat{\sigma }}_{2}\left(Q^{2}\right)\text{cos}\left(2\phi \right)\right\}, \end{array} \end{array}$$ (3)

where $Q^{2}=x_{a}x_{b}S_{\mathrm{pp}}$, with $S_{\mathrm{pp}}$ the center of mass energy squared, and dΩ represents an additional phase space integral. ${\hat{\sigma }}_{0,2}$ are partonic cross-sections calculable perturbatively. Clearly, the $\text{cos}\left(2\phi \right)$ azimuthal asymmetry depends on the spinning gluon NEEC $d_{g,\mathrm{EEC}}$ and ${\hat{\sigma }}_{0,2}$.

The above factorization argument can follow that of Liu and Zhu [5]. A detailed analysis should be carried out in the future, in particular, for the contributions from the Glauber gluons, whose cancellation plays an important role in the factorization at higher orders.

**Fig. 2.**
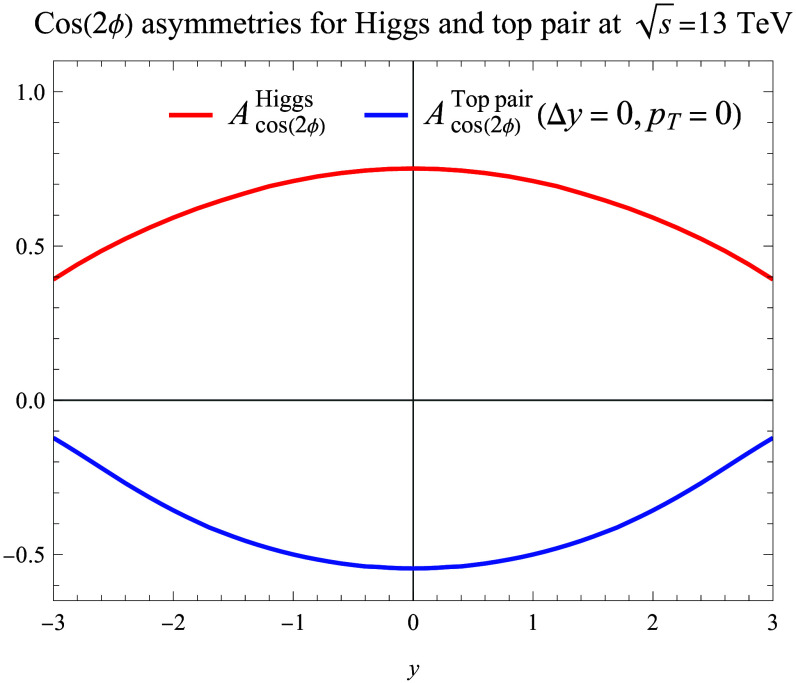
Long-range cos(2ϕ) azimuthal asymmetries associated with Higgs boson production and top quark pair threshold production as functions of their rapidity *y*. The asymmetries are computed from the ratios between the coefficients of cos(2ϕ) and the unpolarized terms in Eq. 3 for both channels.

To study the spinning gluon effect at the LHC, the simplest processes are the Higgs boson production and top quark pair production in pp collisions. We employ perturbative QCD to compute the associated partonic cross-sections ${\hat{\sigma }}_{0,2}$; see Eqs. 6 and 7. In Fig. 2, we show the $\text{cos}\left(2\phi \right)$ asymmetries, the ratios between the coefficients of $\text{cos}\left(2\phi \right)$ and the unpolarized terms in Eq. 3, as functions of rapidity in Higgs boson production and threshold top quark pair production. From this plot, we find that both asymmetries are quite sizable at mid-rapidity, reaching above 50% for both channels. They decrease with rapidity, which reflects the *x* dependence of the spinning gluon and the normal gluon distributions as described in Eqs. 8 and 9. Experimental measurements of these asymmetries will provide important constraints on the gluon spinning effects.

A similar $\text{cos}\left(2\phi \right)$ asymmetry has also been found for Higgs plus two jets’ production, where ϕ is the azimuthal angle between the two jets [61]. In common kinematics, the physics behind these two $\text{cos}\left(2\phi \right)$ is the same, originating from the spinning gluon. In addition, the positive $\text{cos}\left(2\phi \right)$ asymmetry for Higgs boson production is due to its parity. For a CP-odd Higgs, a negative asymmetry would be obtained, similar to those found by Boer et al. [49] and Plehn et al. [61].

**Fig. 3.**
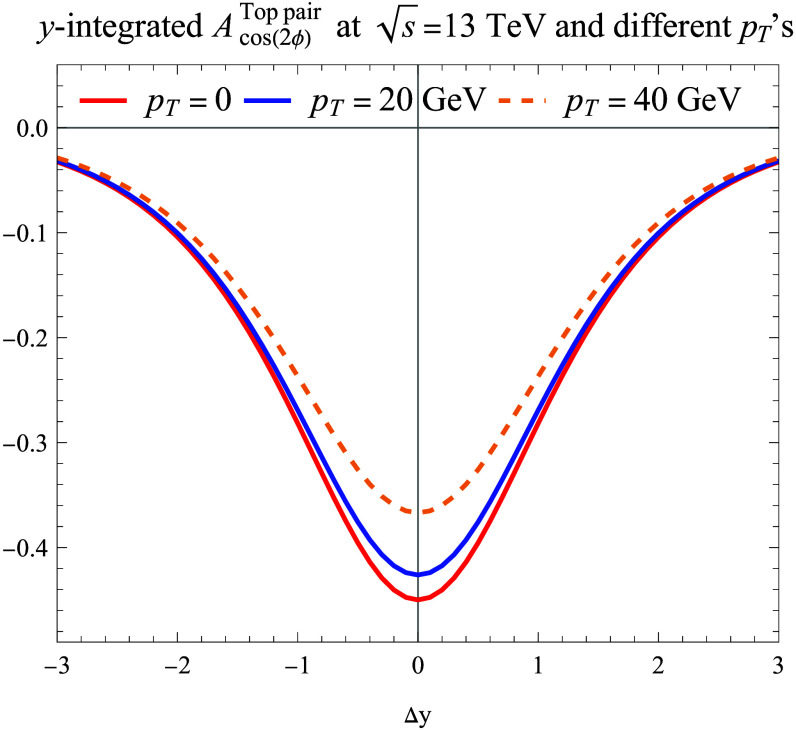
cos(2ϕ) azimuthal asymmetries in the nucleon energy–energy correlator (NEEC) observable associated with top quark pair production as functions of the rapidity difference between the pair Δy at different $p_{T}$ values.

For the top quark pair production, as shown in Fig. 3, the $\text{cos}\left(2\phi \right)$ asymmetry also depends on the top quark transverse momentum and the rapidity difference between the pair $\Delta y=y_{t}-y_{\bar{t}}$ with individual rapidities integrated out. Here, the transverse momentum $p_{T}$ refers to the transverse momentum of an individual quark (or antiquark) in the lab frame, although their total transverse momentum is zero at this order. We have also computed two-photon production through the gluon–gluon fusion process by applying the amplitudes derived in the literature [62–64], and the $\text{cos}\left(2\phi \right)$ asymmetry is smaller as compared to that of Higgs boson production with an opposite sign.

These results demonstrate that the $\text{cos}\left(2\phi \right)$ asymmetries can provide a strong case to study the spinning gluon physics at the LHC. More importantly, this shall open a new avenue to study precision physics in the SM. It may also lead to a unique probe of new physics beyond the SM. In particular, the asymmetries crucially depend on the couplings between the gluon fields with different helicities and the Higgs boson, which have been argued to be sensitive to the new physics beyond the SM, and similar studies on TMD-related observables were carried out by Boer et al. [65,66].

Although the above results are based on leading-order calculations, we expect that higher-order corrections will not modify the large $\text{cos}\left(2\phi \right)$ asymmetries for the above processes. For example, studies on azimuthal asymmetry between the jets in Higgs plus two jets’ production found mild dependence on both higher $\alpha_{s}$ order corrections [67] and parton showers [68]. Therefore, we anticipate this attribute to persist for an NEEC. In view of the higher-order corrections, we notice that the dominant contributions from soft and collinear gluon radiations have the same behavior for $\sigma_{0}$ and $\sigma_{2}$, in particular for those associated with double logarithms at low transverse momentum of Higgs boson production. Therefore, we expect that our main conclusions will remain the same even at higher orders. Of course, a detailed study is needed for a realistic simulation. We will come back to this issue in a later publication.

### Quantum entanglement and test of Bell inequality

The $\text{cos}\left(2\phi \right)$ correlation can be interpreted as a signature of entanglement. In an experiment, what is being measured are the real particles that hit the forward detectors. Although these forward-moving particles never come into contact, they remain entangled in their helicities. The physics picture is as follows: Two pairs of entangled real particles and virtual gluons are created through the splitting of the incoming partons. The virtual gluons will participate the partonic hard process, while the real particles will travel toward the forward detectors at opposite ends of the beam with large momentum $E∼P_{z}\gg P_{t}∼\mathrm{E\theta}$. Once the hard process entangles the virtual gluons, it can be demonstrated that the two real particles become entangled instantaneously. In particular, at the time when they are produced, the helicity states of the 2 forward (backward) propagating real partons, $p_{a}$ and $p_{b}$, from the independent splitting processes $\left(p_{\alpha },\to ,p_{a},g_{a}^{*}\right.$ and $\left.p_{\beta },\to ,p_{b},g_{b}^{*}\right)$ are separable, where $p_{\alpha }$ and $p_{\beta }$ represent the partons before the splitting. However, since the hard interaction will entangle the virtual gluons to have a helicity state of $|g_{a}^{*}g_{b}^{*}\rangle \propto |++\rangle +|--\rangle$, it means that if $g_{a}^{*}$ is with +-helicity, then $g_{b}^{*}$ has also to be in +-helicity. This will in turn force $p_{a}$ and $p_{b}$ to be entangled although they never come into interact with each other directly.

This observation provides a basis for testing Bell’s theorem [8] through the $\text{cos}\left(2\phi \right)$ correlation. Leveraging the NEEC in Eq. 3, one can formulate the Bell observable [69,70]:

$$\begin{array}{c} S\left(\phi_{a},\;\phi_{b}\right)\equiv \frac{\Sigma \left(\phi_{a},\;\phi_{b}\right)+\Sigma \left(\phi_{a}^{\prime },\;\phi_{b}^{\prime }\right)-\Sigma \left(\phi_{a}^{\prime },\;\phi_{b}\right)-\Sigma \left(\phi_{a},\;\phi_{b}^{\prime }\right)}{\Sigma \left(\phi_{a},\;\phi_{b}\right)+\Sigma \left(\phi_{a}^{\prime },\;\phi_{b}^{\prime }\right)+\Sigma \left(\phi_{a}^{\prime },\;\phi_{b}\right)+\Sigma \left(\phi_{a},\;\phi_{b}^{\prime }\right)}, \end{array}$$ (4)

where $\phi_{a}$ and $\phi_{b}$ are azimuthal angles of the energy flow directed toward the detector, measured with respect to arbitrary reference vectors $r_{a,b}$. $\phi^{\prime }=\phi +\pi /2$ and can be regarded as one measures the azimuthal angles with the reference vectors perpendicular to $r_{a,b}$. For appropriate choices of $\phi_{a,b}$ and ${\tilde{\phi }}_{a,b}$, the Clauser–Horne–Shimony–Holt (CHSH) inequality [9], an equivalent version of the Bell’s original inequality,

$$\begin{array}{c} B\equiv \left|S\left(\phi_{a},\;\phi_{b}\right)-S\left(\phi_{a},\;{\tilde{\phi }}_{b}\right)+S\left({\tilde{\phi }}_{a},\;\phi_{b}\right)+S\left({\tilde{\phi }}_{a},\;{\tilde{\phi }}_{b}\right)\right|\leq 2, \end{array}$$ (5)

can potentially be violated. The maximum violation of the CHSH inequality for any quantum state is given by the Tsirelson’s bound, $B_{\max}=2\sqrt{2}\approx 2.828$ [71]. A proof of Eq. 5 can be found in the Supplementary Materials.

**Fig. 4.**
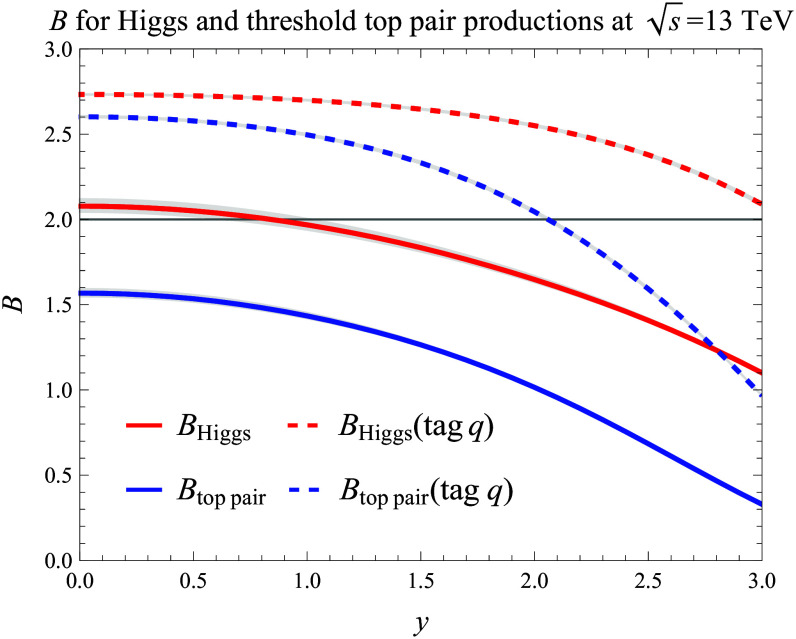
Violation of the Clauser–Horne–Shimony–Holt (CHSH) inequality in Higgs (red) and top pair (blue) production at the LHC. Quark jet tagging (dashed lines) substantially enhances the significance.

Figure 4 demonstrates the concept by measuring the CHSH inequality in Eq. 5 using the NEEC factorization in Eq. 3. We choose $\phi_{a}=0$, $\phi_{b}=\pi /8$, ${\tilde{\phi }}_{a}=\pi /4$, and ${\tilde{\phi }}_{b}=3\pi /8$ [71]. Violation of the CHSH inequality is observed for the Higgs rapidity $y_{\text{Higgs}}<0.5$. We note that the significance can be dramatically improved by quark jet tagging, meaning that the forward and backward detection of quarks and the NEEC gluon distributions in Eqs. 4 and 5 only receive contributions from the quark splittings, as manifest from Fig. 4, where the CHSH inequality violation is observed for both Higgs and $t\bar{t}$ threshold production. Experimentally, this will be a great challenge and we hope that our results in Fig. 4 will motivate further developments. We also check that increasing the machine energy leads to a more substantial violation, reaching B≈2.36 for $y_{\text{Higgs}}=0$ at $\sqrt{S_{\mathrm{pp}}}=33\;\mathrm{TeV}$ without jet tagging, as the entanglement between the detected forward-moving particles intensifies near small *x* values.

## Conclusion

In summary, we studied the long-range azimuthal angular correlations in NEEC measurements in pp collisions at the LHC. For a number of processes, we found substantial large $\text{cos}\left(2\phi \right)$ asymmetries. The comparison between these and future studies at the electron–ion collider will provide an important test of the universality of the NEEC distribution functions. Because of the large asymmetries in these processes at the LHC, we emphasize that this will also open a new avenue to study precision physics for the SM, in particular through comparison between Higgs boson production, top quark pair production, and two-photon production. Of course, toward this goal, the backgrounds from other channels are important to explore as well. For example, for Higgs boson production process, there is a weak boson fusion contribution. Although the weak boson fusion contribution is an order of magnitude smaller than the gluon fusion contribution in the total rate, it can potentially dilute the signal. A future study on this should be pursued to solidify the signal.

Although the partonic processes, as those studied in this paper, in general are intrinsically quantum, the quantum entanglement is not always manifest in physical observables. The connection between the $\text{cos}\left(2\phi \right)$ correlation and the entanglement makes the long-range correlation in NEEC a promising channel to investigate the quantum entanglement and provide a fundamental test of Bell’s inequality. We demonstrate the feasibility of this approach using Higgs and threshold $t\bar{t}$ production at the LHC in which violation of the Bell inequality is substantial when we perform quark jet tagging. Compared to the other collider-based tests discussed in the literature [10–15,17–21,31–33], the long-range correlation in NEEC enables, for the first time, a test of this fundamental quantum property in confined quantities like gluons. Our method benefits from the NEEC factorization theorem, ensuring that the test remains local, thus closing the major potential loophole [32] present in LHC-based tests. Moreover, unlike previous proposals that often require reconstructing the full kinematics, which is usually challenging at the LHC, the NEEC measurement only requires determining the azimuthal angles of the energy flow deposit at the forward detectors, making it more practical for experimental implementation.

Looking ahead, extending this research to other QCD processes, including multijet production, and heavy quarkonium production, will be interesting to follow. Additionally, recent investigations [72–82] have indicated that the quantum entanglement may bring novel perspectives into nuclear and particle physics. We thus anticipate that our work may spark similar endeavors in unraveling the nucleon structures using the quantum information properties. These studies will promise to yield deeper insights into the effects of spinning gluons, complement our current understanding, and potentially reveal new physics beyond the SM.

## Methods

**Fig. 5.**
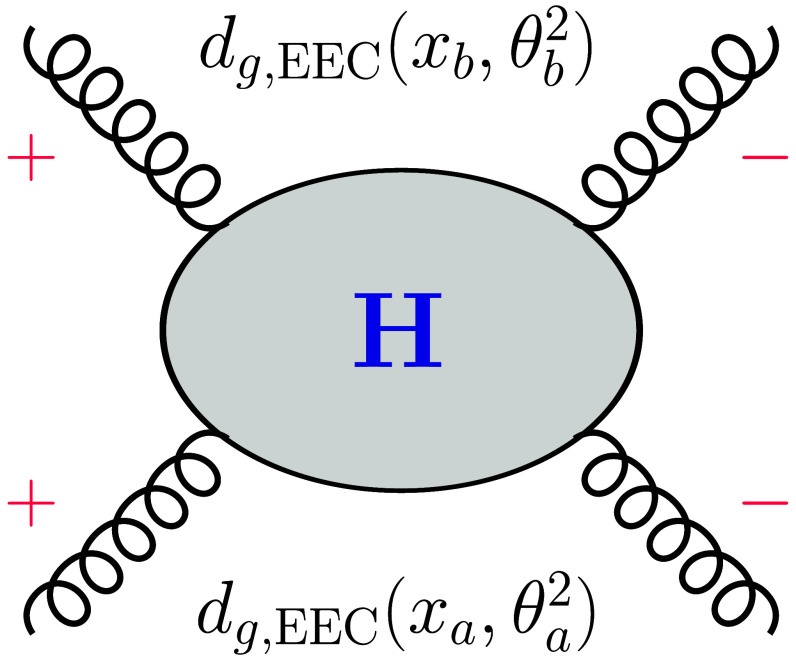
Long-range cos(2ϕ) asymmetry comes from the interference between double helicity-flip amplitudes in the partonic scattering processes.

To derive the $\text{cos}\left(2\phi \right)$ asymmetry in Eq. 3 for the hard processes in pp collisions at the LHC, we apply perturbative QCD to compute the partonic cross-sections ${\hat{\sigma }}_{0,2}$. In particular, the $\text{cos}\left(2\phi \right)$ term ${\hat{\sigma }}_{2}$ comes from the interference between double helicity-flip amplitudes where both incoming gluons have the same helicity as illustrated in Fig. 5. In this paper, we focus on the Higgs boson production and top quark pair production processes at the LHC.

For the Higgs boson production process, similar to the TMD case calculated before [48,65,83], the Higgs boson can couple to the spinning gluons directly, and at the leading order

$${\hat{\sigma }}_{2}={\hat{\sigma }}_{0}=\pi g_{\phi }^{2}/64,$$ (6)

where $g_{\phi }$ represents the coupling between the Higgs boson and the gluon fields in the effective theory $L_{\mathrm{eff}}=-\left(1/4\right)g_{\phi }\Phi F_{\mu \nu }^{a}F^{\mathrm{a\mu \nu}}$ [84]. The above shows that the $\text{cos}\left(2\phi \right)$ asymmetry for Higgs production is positive and can reach a sizable value depending on the ratio between $d_{g,\mathrm{EEC}}$ and $f_{g,\mathrm{EEC}}$. On the other hand, for the top quark pair production, ${\hat{\sigma }}_{2}$ is different from ${\hat{\sigma }}_{0}$,

$$\begin{array}{c} \begin{array}{l} {\hat{\sigma }}_{0}=\frac{\alpha_{s}^{2}\pi }{{\hat{s}}^{2}}\left[\frac{1}{6}\frac{1}{{\hat{t}}_{1}{\hat{u}}_{1}}-\frac{3}{8}\frac{1}{{\hat{s}}^{2}}\right]\left[{\hat{t}}_{1}^{2}+{\hat{u}}_{1}^{2}+4m_{t}^{2}\hat{s}-\frac{4m_{t}^{4}{\hat{s}}^{2}}{{\hat{t}}_{1}{\hat{u}}_{1}}\right],\\ {\hat{\sigma }}_{2}=-\frac{\alpha_{s}^{2}\pi }{{\hat{s}}^{2}}\left[\frac{1}{6}\frac{1}{{\hat{t}}_{1}{\hat{u}}_{1}}-\frac{3}{8}\frac{1}{{\hat{s}}^{2}}\right]\frac{2m_{t}^{4}{\hat{s}}^{2}}{{\hat{t}}_{1}{\hat{u}}_{1}}, \end{array} \end{array}$$ (7)

for the dominant $\mathrm{gg}\to t\bar{t}$ channel, where ${\hat{t}}_{1}=\hat{t}-m_{t}^{2}$ and ${\hat{u}}_{1}=\hat{u}-m_{t}^{2}$ and $\hat{s}$, $\hat{t}$, and $\hat{u}$ are the usual Mandelstam variables. In contrast to the Higgs case, the $\text{cos}\left(2\phi \right)$ asymmetry for top quark pair production is negative. Interestingly, the asymmetry will reach the maximum value when the pair are close to the threshold where $\hat{s}=4m_{t}^{2}$.

Of course, the final results of $\text{cos}\left(2\phi \right)$ asymmetries also depend on the NEEC gluon distributions. When $P^{+}\theta_{a}\gg \Lambda_{\mathrm{QCD}}$, they can be computed from perturbative QCD with collinear splitting contributions,

$$\begin{array}{l} f_{g,\mathrm{EEC}}\left(x,\;\theta_{a}^{2}\right)=\frac{\alpha_{s}}{2\pi }\frac{1}{\theta_{a}^{2}}\int_{x}^{1}\frac{\mathrm{dz}}{z}\frac{x\left(1-z\right)}{z}\\ \times \left[P_{g/q}\left(z\right)f_{q}\left(\frac{x}{z}\right)+P_{g/g}\left(z\right)f_{g}\left(\frac{x}{z}\right)\right], \end{array}$$ (8)

$$\begin{array}{l} d_{g,\mathrm{EEC}}\left(x,\;\theta_{a}^{2}\right)=\frac{\alpha_{s}}{2\pi }\frac{1}{\theta_{a}^{2}}\int_{x}^{1}\frac{\mathrm{dz}}{z}\frac{x\left(1-z\right)}{z}\\ \times \frac{2\left(1-z\right)}{z}\left[C_{F}f_{q}\left(\frac{x}{z}\right)+C_{A}f_{g}\left(\frac{x}{z}\right)\right], \end{array}$$ (9)

where $P_{g/q}$ and $P_{g/g}$ are the usual collinear splitting kernels. It is interesting to note that the quark splitting contribution to the spinning gluon $d_{g,\mathrm{EEC}}$ leads to the same sign as the gluon splitting one. This is different from the fragmentation case in Chen et al. [56], where there is a cancellation between quark and gluon splitting contributions. Additional Dokshitzer–Gribov–Lipatov–Altarelli–Parisi resummation will modify the power behavior, for which we expect a similar effect for both $f_{g,\mathrm{EEC}}$ and $d_{g,\mathrm{EEC}}$ [56].

## Data Availability

All data are available in the main text.

## Supplementary Materials

Supplementary 1 Proof of the CHSH inequality in Eq. 9
